# Association of Missense Polymorphism in *HSD3B1* With Outcomes Among Men With Prostate Cancer Treated With Androgen-Deprivation Therapy or Abiraterone

**DOI:** 10.1001/jamanetworkopen.2019.0115

**Published:** 2019-02-22

**Authors:** Masaki Shiota, Shintaro Narita, Shusuke Akamatsu, Naohiro Fujimoto, Takayuki Sumiyoshi, Maki Fujiwara, Takeshi Uchiumi, Tomonori Habuchi, Osamu Ogawa, Masatoshi Eto

**Affiliations:** 1 Department of Urology, Graduate School of Medical Sciences, Kyushu University, Fukuoka, Japan; 2 Department of Urology, Graduate School of Medicine, Akita University, Akita, Japan; 3 Department of Urology, Graduate School of Medicine, Kyoto University, Kyoto, Japan; 4 Department of Urology, School of Medicine, University of Occupational and Environmental Health, Kitakyushu, Japan; 5 Department of Clinical Chemistry and Laboratory Medicine, Graduate School of Medical Sciences, Kyushu University, Fukuoka, Japan

## Abstract

**Importance:**

Recently, genetic polymorphism in *HSD3B1* encoding 3β-hydroxysteroid dehydrogenase-1 has been shown to be associated with oncological outcome when treated with androgen-deprivation therapy (ADT) for prostate cancer. Upfront abiraterone combined with ADT has proved survival benefit. However, its effect on oncological outcome among different ethnicities and in abiraterone treatment remain unclear.

**Objective:**

To investigate the significance of missense polymorphism in *HSD3B1* gene among men treated with primary ADT or abiraterone.

**Design, Setting, and Participants:**

This prognostic study included Japanese patients with metastatic hormone-sensitive prostate cancer between June 1993 and July 2005 and with castration-resistant prostate cancer between September 2014 and February 2018. Genome DNA was obtained from patient whole blood samples, and genotyping on *HSD3B1* (rs1047303, 1245C) was performed by Sanger sequencing.

**Exposures:**

Primary ADT for metastatic hormone-sensitive prostate cancer and abiraterone for castration-resistant prostate cancer.

**Main Outcomes and Measures:**

The association of genotype in *HSD3B1* with clinicopathological parameters and oncological outcome, including prostate-specific antigen response, progression-free survival, treatment failure–free survival, and overall survival was examined.

**Results:**

Of 203 men, 104 were in the primary ADT cohort (median [interquartile range] age, 72 [67-76] years) and 99 men were in the abiraterone group (median [interquartile range] age, 74 [67-80] years). Most patients carried metastatic lesions in each cohort. Among the cohort of primary ADT, men carrying heterozygous and homozygous variant types in *HSD3B1* gene showed higher progression risk (hazard ratio [HR], 2.34; 95% CI, 1.08-4.49; *P* = .03) but not any-caused death risk (HR, 1.36; 95% CI, 0.52-2.92; *P* = .50), compared with men carrying homozygous wild type. In contrast, among the abiraterone cohort, men carrying variant type in *HSD3B1* gene showed lower progression risk (HR, 0.32; 95% CI, 0.12-0.69; *P* = .006) and lower all-cause mortality risk (HR, 0.40; 95% CI, 0.13-0.94; *P* = .04) compared with men carrying homozygous wild type.

**Conclusions and Relevance:**

This study showed that *HSD3B1* genetic variant is distinctly associated with oncological outcome between primary ADT and abiraterone in Japanese men, suggesting universal significance among different ethnicities in primary ADT, as well as promise as a predictive biomarker of ADT and abiraterone.

## Key Points

**Question:**

What is the clinical impact of genetic variant in *HSD3B1* when treated with androgen-deprivation therapy (ADT) and abiraterone for prostate cancer?

**Findings:**

In this prognostic study of 203 Japanese men, the prognosis in variant carriers of *HSD3B1* was worse in the ADT group among 104 men with metastatic hormone-sensitive prostate cancer. However, variant carriers of *HSD3B1* among 99 men with castration-resistant prostate cancer showed distinctly better response to abiraterone therapy.

**Meaning:**

Genotype in *HSD3B1* may serve as a promising biomarker for ADT and abiraterone, suggesting an application for upfront abiraterone with ADT for individuals with hormone-sensitive prostate cancer.

## Teaser

This prognostic study investigates the significance of missense polymorphism in the *HSD3B1 *gene among men treated with primary androgen-deprivation therapy or abiraterone for prostate cancer.

## Introduction

Androgens play critical roles in prostate carcinogenesis as well as prostate cancer progression. Since 1941, androgen-deprivation therapy (ADT), which reduces testosterone production and inhibits androgen action in prostate cancer cells, has been the criterion standard therapy for metastatic prostate cancer.^1^ Although initially most prostate cancers respond well to ADT, most patients eventually progress to castration-resistant prostate cancer (CRPC), which is mainly thought to be because androgen receptor reactivation is induced by several mechanisms.^2^ One of those mechanisms has been identified to be intratumoral androgen synthesis mostly from adrenal precursor steroids and at least in part due to de novo synthesis from cholesterol,^3,4^ which is supported by increased expression of several genes encoding steroidogenic enzymes including *HSD3B*, *HSD17B*, and *SRD5A* in CRPC.^5^ Among them, *HSD3B1* encodes 3β-hydroxysteroid dehydrogenase-1, which is mainly expressed in peripheral tissues including the prostate, breast, skin, and placenta (another isoform, 3β-hydroxysteroid dehydrogenase-2 was mainly expressed in adrenal gland and gonad in human) and is a rate-limiting enzyme required for all pathways of dihydrotestosterone synthesis.^6^ Recently, a mutation (1245A→C) in *HSD3B1* was shown to provide a novel mechanism of resistance to ADT,^7^ where amino acid 367 Asn→Thr is changed and 3β-hydroxysteroid dehydrogenase-1 is rendered to be resistant to proteasomal degradation, causing substantial accumulation of this enzyme and gain of function. Although the *HSD3B1* (1245C) allele can be acquired by mutation, germ-line single-nucleotide polymorphism (rs1047303) is also known to exist.

Recently, upfront abiraterone in combination with primary ADT has been shown to improve survival for metastatic hormone-sensitive prostate cancer (HSPC).^8,9^ However, it remains unclear who is suitable for upfront abiraterone therapy to metastatic HSPC. Intriguingly, it has been reported that abiraterone is converted by 3β-hydroxysteroid dehydrogenase to Δ^4^-abiraterone (D4A), which blocks multiple steroidogenic enzymes and antagonizes the androgen receptor, providing an additional explanation for clinical activity by abiraterone.^10^ Therefore, tumors in men carrying variant genotype in *HSD3B1* showing higher enzymatic activity of 3β-hydroxysteroid dehydrogenase-1 may be vulnerable to abiraterone owing to higher concentration of D4A.

Recent studies have demonstrated that genetic polymorphism in *HSD3B1* is associated with oncological outcome among residents in the United States treated with ADT, where men carrying variant alleles showed worse prognosis.^11,12,13^ Thus, genetic variation in *HSD3B1* (1245C) genotype is a promising predictive biomarker of positive ADT response among men with prostate cancer. However, its impact on prognosis among people of different ethnicities remains unclear, where the frequency of the variant allele would differ among ethnicities. In addition, the significance of *HSD3B1* genotype in abiraterone treatment is rarely investigated. Accordingly, in this study, we aimed to investigate the association between genetic variants in *HSD3B1* (1245C) and oncological outcome in Japanese men treated with primary ADT for metastatic HSPC as well as abiraterone for CRPC.

## Methods

### Patients

Japanese patients who had undergone primary ADT for metastatic HSPC to regional lymph nodes or distant sites at the University of Occupational and Environmental Health (Kitakyushu, Japan) and Kyushu University Hospital (Fukuoka, Japan) between June 1993 and July 2005 were consecutively included in the cohort of primary ADT.^14,15,16^ All patients were histopathologically diagnosed as having adenocarcinoma of the prostate. Clinical TNM staging was determined in accordance with the unified TNM criteria based on the results of digital rectal examination, transrectal ultrasound, magnetic resonance imaging, computed tomography, and bone scan.^17^ All patients were primarily treated with surgical castration or medical castration using a gonadotropin-releasing hormone agonist (goserelin acetate or leuprorelin acetate) with or without an antiandrogen agent (bicalutamide, flutamide, or chlormadinone acetate). Progressive disease was defined as an increase in serum prostate-specific antigen (PSA) levels of more than 2 ng/mL and a 25% increase over the nadir, the appearance of a new lesion, or the progression of 1 or more known lesions classified according to the Response Evaluation Criteria in Solid Tumors.^18^

Japanese patients who were treated with abiraterone for CRPC at the Akita University Hospital (Akita, Japan), Kyoyo University Hospital (Kyoto, Japan), and Kyushu University Hospital (Fukuoka, Japan) between September 2014 and February 2018 were included in the abiraterone cohort. Clinical staging was determined based on the results of computed tomography and bone scan.^17^ Patients were treated with 1000 mg of abiraterone plus 10 mg of prednisolone daily with surgical castration or medical castration using a gonadotropin-releasing hormone agonist/antagonist (goserelin acetate, leuprorelin acetate, or degarelix acetate). Prostate-specific antigen response was defined as maximum decline of PSA after abiraterone administration. Treatment failure was defined as radiological progression including the appearance of a new metastatic lesion, the progression of 1 or more known lesions classified according to the Response Evaluation Criteria in Solid Tumors,^17^ or discontinuation of abiraterone owing to no clinical benefit judged by physicians.

Written informed consent was obtained from all patients. The patients who declined to be included in this study were excluded. Informed consent and blood samples were obtained before the initiation of therapy. This study was performed in accordance with the principles described in the Declaration of Helsinki^19^ and the Ethical Guidelines for Epidemiological Research enacted by the Japanese government and approved by each institutional review board. Reporting followed the Transparent Reporting of a Multivariable Prediction Model for Individual Prognosis or Diagnosis (TRIPOD) reporting guideline.^20^

### Genotyping

Genomic DNA was extracted from patient whole blood samples. *HSD3B1* (rs1047303) genotyping was performed by sequencing as described previously.^21^ Briefly, pathologic complete response amplification was performed using TaKaRa EmeraldAmp PCR Master Mix (TaKaRa). The primers, annealing temperature, and cycle numbers were as follows: 5′-GTCAAATAGCGTATTCACCTTCTCTTAT-3′ and 5′-GAGGGTGGAGCTTGATGACATCT-3′, annealing temperature: 65°C, 35 cycles, respectively. The pathologic complete response products were purified using the TaKaRa NucleoSpin Gel and PCR clean-up (TaKaRa) and sequenced using the BigDye Terminator version 3.1 Cycle Sequencing Kit (Applied Biosystems) on a Genetic Analyzer 3130XL (Applied Biosystems). Sequence data were visualized using Sequence Scanner Software version 1.0 (Applied Biosystems).

### Statistical Analysis

All statistical analyses were performed using JMP software, version 13 (SAS Institute). Categorical and continuous data were analyzed by Pearson χ^2^ and Wilcoxon rank sum tests, respectively. Survival analyses were conducted using the Kaplan-Meier method and the log-rank test. Univariate analyses were performed using the Cox hazard proportional model to estimate hazard ratios (HRs and 95% CIs). All *P* values were 2-sided. *P* values less than .05 were considered significant.

## Results

### Significance of Genetic Variation in *HSD3B1* in Primary ADT

Clinical and pathological characteristics of 104 Japanese patients in the primary ADT cohort are displayed in eTable 1 in the Supplement. The median patient age was 72 years (interquartile range [IQR], 67-76 years; range, 44-87 years), and the median PSA at diagnosis was 244.0 ng/mL (IQR, 85.5-744.3 ng/mL). The Gleason scores of biopsy specimens from 31 patients (32.6%) and 64 patients (67.4%) were less than 8 and 8 or higher, respectively. The clinical T-stages were cT2/3 and cT4 in 65 patients (72.2%) and 25 patients (27.8%), respectively. Metastasis to the regional lymph nodes (N1) and distant sites (M1) were detected in 51 patients (56.0%) and 94 patients (90.4%), respectively. Twelve patients (11.5%) were primarily treated with castration alone and 92 patients (88.5%), with combined androgen blockade. During a median follow-up of 3.7 years (IQR, 1.6-7.6 years), disease progression and any caused death occurred in 85 cases (81.7%) and 60 cases (57.7%), respectively. The median progression-free survival (PFS) and overall survival (OS) were 1.2 years and 6.0 years, respectively.

First, associations between genetic polymorphisms in *HSD3B1* and clinicopathological parameters were analyzed. Homozygous wild type, heterozygous variant type, and homozygous variant type were detected in 95 men (91.3%), 7 men (6.7%), and 2 men (1.9%), respectively. Among them, no differences in clinicopathological parameters including PSA at diagnosis, Gleason score, and clinical stage were observed (eTable 1 in the Supplement).

**Figure 1.**
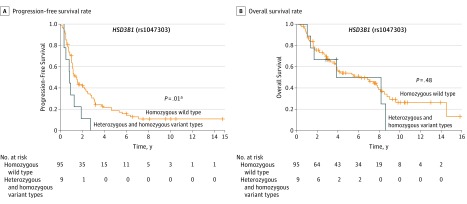
Association of Gene Polymorphism in *HSD3B1* rs1047303 With Prognosis in Cases With Metastatic Hormone-Sensitive Prostate Cancer Treated With Primary Androgen-Deprivation Therapy Progression-free survival rate (A) and overall survival rate (B) stratified by gene polymorphism in *HSD3B1* rs1047303 are shown. ^a^Statistically significant.

Next, we analyzed the prognostic impact of *HSD3B1* genotype status on PFS and OS. Using univariate analyses, heterozygous and homozygous variant types were significantly associated with higher risk of progression (HR, 2.16; 95% CI, 1.14-3.85; *P* = .02) compared with homozygous wild type (Table 1). Kaplan-Meier analyses demonstrated an advantage in PFS of men with *HSD3B1* homozygous wild type status compared with men carrying heterozygous and homozygous variant types (Figure 1A). However, OS was comparable between men with homozygous wild type and men with heterozygous and homozygous variant types (HR, 1.3; 95% CI, 0.52-2.92; *P* = .50) as shown with Kaplan-Meier curve (Figure 1B).

**Table 1.** Associations Between Clinicopathological Parameters and Prognosis in Primary ADT

Variable	Progression-Free Survival		Overall Survival	
	HR (95% CI)	*P* Value	HR (95% CI)	*P* Value
Age, HR (range), y	1.56 (0.49-5.26)	.46	3.99 (0.98-17.53)	.05
PSA at diagnosis (range)	2.94 (0.69-9.33)	.13	1.00 (0.11-5.78)	>.99
Biopsy Gleason score				
<8	1 [Reference]	NA	1 [Reference]	NA
≥8	1.67 (1.03-2.77)	.04^a^	1.25 (0.72-2.28)	.44
Clinical T-stage				
cT2/3	1 [Reference]	NA	1 [Reference]	NA
cT4	1.20 (0.70-1.97)	.50	1.49 (0.79-2.67)	.21
Clinical N-stage				
N0	1 [Reference]	NA	1 [Reference]	NA
N1	1.30 (0.82-2.07)	.26	1.08 (0.62-1.89)	.77
Clinical M-stage				
M0	1 [Reference]	NA	1 [Reference]	NA
M1	1.67 (0.79-4.31)	.19	1.83 (0.74-6.10)	.21
Hormonal therapy				
Combined androgen blockade	1 [Reference]	NA	1 [Reference]	NA
Castration	0.74 (0.36-1.37)	.35	1.07 (0.44-2.21)	.86
*HSD3B1* (rs1047303)				
Homozygous wild type	1 [Reference]	NA	1 [Reference]	NA
Heterozygous and homozygous variant types	2.34 (1.08-4.49)	.03^a^	1.36 (0.52-2.92)	.50

Abbreviations: ADT, androgen-deprivation therapy; HR, hazard ratio; NA, not applicable; PSA, prostate-specific antigen.

^a^ Statistically significant.

### Significance of Genetic Variation in *HSD3B1* in Abiraterone

Clinical and pathological characteristics of 99 Japanese patients in the abiraterone cohort are displayed in eTable 2 in the Supplement. The median patient age was 74 years (IQR, 67-80 years). The Gleason scores of biopsy specimens from 14 patients (15.4%) and 77 patients (84.6%) were less than 8 and 8 or higher, respectively. The median PSA at pretreatment of abiraterone was 14.7 ng/mL (IQR, 4.7-87.1 ng/mL). The clinical M-stages were M0, M1a, M1b, and M1c in 8 patients (8.1%), 10 patients (10.1%), 68 patients (68.7%), and 13 patients (13.1%), respectively. Prior enzalutamide and prior docetaxel were administered in 46 patients (46.5%) and 41 patients (41.4%), respectively. During a median follow-up of 1.2 years (IQR, 0.8-1.9 years), treatment failure occurred in 75 patients (75.8%), and all-cause mortality occurred in 50 patients (50.5%). The median time to treatment failure was 0.5 years, and median OS was 1.8 years.

First, associations between genetic polymorphisms in *HSD3B1* and clinicopathological parameters were analyzed. Homozygous wild type and heterozygous variant type were detected in 85 men (85.9%) and 14 men (14.1%), respectively, while homozygous variant type was not detected in this cohort. Among them, prior enzalutamide was more frequent in homozygous wild type although no differences in other clinicopathological parameters were observed (eTable 2 in the Supplement).

**Figure 2.**
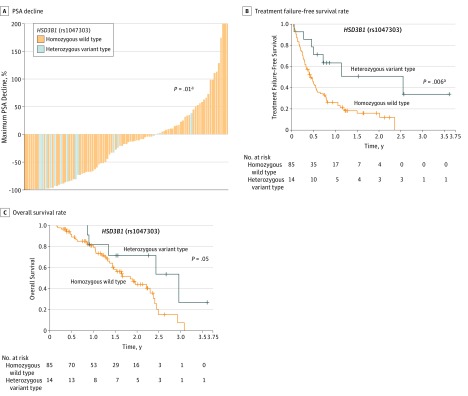
Association of Gene Polymorphism in *HSD3B1* rs1047303 With Sensitivity and Prognosis in Cases With Castration-Resistant Prostate Cancer Treated With Abiraterone A, Waterfall plots showing the greatest decline in prostate-specific antigen (PSA) values from baseline during abiraterone treatment in 97 men whose PSA response were available. B and C, Treatment failure–free survival rate (B) and overall survival rate (C) stratified by gene polymorphism in *HSD3B1* rs1047303 are shown. ^a^Statistically significant.

Next, we analyzed clinical significance of genetic polymorphism in *HSD3B1* on PSA response, treatment failure–free survival, and OS. Maximum PSA declines during abiraterone treatment in heterozygous variant type (median, −83.4%; IQR, −97.4% to −18.7%) were significantly superior to those in homozygous wild type (median, −12.2%; IQR, −67.9% to 25.3%; *P* = .01) (Figure 2A). Using univariate analyses, heterozygous variant type was significantly associated with lower risk of treatment failure (HR, 0.32; 95% CI, 0.12-0.69; *P* = .002) compared with homozygous wild type (Table 2). Kaplan-Meier curve distinguished treatment failure–free survival by genetic polymorphism in *HSD3B1* between men carrying homozygous wild type and men carrying heterozygous variant type (Figure 2B). When adjusted with PSA at pretreatment, clinical M-stage, prior enzalutamide, and prior docetaxel using univariate analyses, heterozygous variant type was significantly associated with lower risk of treatment failure (HR, 0.35; 95% CI, 0.13-0.80; *P* = .01) compared with homozygous wild type. Similarly, heterozygous variant type was significantly associated with lower risk of all-cause mortality (HR, 0.40; 95% CI, 0.13-0.94; *P* = .04) compared with homozygous wild type (Table 2), as shown with Kaplan-Meier curve (Figure 2C). When adjusted with PSA at pretreatment, clinical M-stage, prior enzalutamide, and prior docetaxel using multivariate analyses, the significance of genetic polymorphism in *HSD3B1* was not relevant with all-cause mortality (HR, 0.48; 95% CI, 0.16-1.25; *P* = .14).

**Table 2.** Associations Between Clinicopathological Parameters and Prognosis in Abiraterone

Variable	Treatment Failure–Free Survival		Overall Survival	
	HR (95% CI)	*P* Value	HR (95% CI)	*P* Value
Age at pretreatment, HR (range), y	0.80 (0.24-2.76)	.72	2.09 (0.40-10.87)	.38
PSA at diagnosis, HR (range)	0.52 (0.047-2.54)	.47	0.33 (0.0017-4.57)	.50
Biopsy Gleason score				
<8	1 [Reference]	NA	1 [Reference]	NA
≥8	1.14 (0.45-1.58)	.67	0.89 (0.45-1.99)	.77
PSA at pretreatment, HR (range)	9.88 (2.85-27.11)	.001^a^	50.20 (12.26-191.36)	<.001^a^
ECOG PS at pretreatment				
0	1 [Reference]	NA	1 [Reference]	NA
1	1.48 (0.88-2.43)	.14	1.15 (0.60-2.14)	.67
≥2	1.89 (0.77-3.99)	.15	2.00 (0.78-4.48)	.14
Clinical M-stage				
M0	0.87 (0.30-1.99)	.86	1.18 (0.19-3.97)	.83
M1a	0.79 (0.32-1.63)	.54	0.46 (0.075-1.55)	.24
M1b	1 [Reference]	NA	1 [Reference]	NA
M1c	2.23 (1.09-4.21)	.03^a^	2.96 (1.24-6.29)	.02^a^
Prior enzalutamide				
Absence	1 [Reference]	NA	1 [Reference]	NA
Presence	3.51 (2.15-5.81)	<.001^a^	2.23 (1.25-4.05)	.007^a^
Prior docetaxel				
Absence	1 [Reference]	NA	1 [Reference]	NA
Presence	1.87 (1.18-2.97)	.008^a^	1.34 (0.75-2.40)	.32
*HSD3B1* (rs1047303)				
Homozygous wild type	1 [Reference]	NA	1 [Reference]	NA
Heterozygous variant type	0.32 (0.12-0.69)	.002^a^	0.40 (0.13-0.94)	.04^a^

Abbreviations: ECOG, Eastern Cooperative Oncology Group; HR, hazard ratio; NA, not applicable; PS, performance status; PSA, prostate-specific antigen.

^a^ Statistically significant.

## Discussion

Previous studies investigating the effect of *HSD3B1* on oncological outcome have demonstrated higher risk of progression and all-cause mortality in men carrying the variant allele.^11,12,13^ Although ethnic distribution in the study by Agarwal et al^12^ is not presented, the study by Hearn et al^11^ included mainly white men. In contrast to these US reports, the Chinese study by Wu et al^22^ showed significant risk of CRPC in men carrying variant allele but failed to show significant differences in PFS and OS. Thus, to our knowledge, for the first time in Asian individuals, the current study clearly shows significant detrimental PFS associated with variant allele. Superior quality of the sample origin might contribute to the successful demonstration of the significant results in this study, contrary to the results of the Chinese study by Wu et al^22^ that used samples obtained from formalin-fixed archival tissues, which may cause errors in sequencing and may contain tumor-derived DNA. In addition, an imbalance in patients with nonmetastasis and metastasis between genotypes may explain the reason of failure to show prognostic significance in the study by Wu et al.^22^ Higher frequency in the current study of variant allele in CRPC (14.1%) compared with that in HSPC (8.7%) also supports the hypothesis that men carrying variant allele tend to become resistant to ADT. However, the current study failed to show significant results in OS, contrary to the findings of the US study by Hearn et al,^11^ which may be due to the rare frequency of variant type in Japanese men or subsequent therapy such as abiraterone for CRPC.

Intriguingly, this study showed opposite oncological outcomes in abiraterone treatment among men carrying variant type, in contrast to those in primary ADT. Abiraterone was shown to be converted into more active D4A by 3β-hydroxysteroid dehydrogenase-1^10^ and further into paradoxically androgen receptor agonist 3-keto-5α-abiraterone by 5α-reductase as 1 of 6 metabolites from D4A.^23^ Then, it may theoretically be reasonable that men with variant type in *HSD3B1* with higher activity of 3β-hydroxysteroid dehydrogenase-1 showed favorable response and prognosis in abiraterone treatment. Thus, increased metabolism of abiraterone into more active D4A leading to better therapeutic effect of abiraterone may be achieved in men with higher activity of 3β-hydroxysteroid dehydrogenase-1, while extragonadal androgen synthesis leading to progression to CRPC may be augmented. Moreover, opposite prognostic impact of *HSD3B1* genotype indicated promise as a predictive biomarker for ADT and abiraterone treatment. Recently, upfront abiraterone treatment was shown to improve survival in metastatic hormone-sensitive prostate cancer by the LATITUDE^8^ and STAMPEDE^9^ trials although a predictive biomarker was not identified so far. According to the result in the current study, men carrying variant type in *HSD3B1* gene may be suitable candidates for upfront abiraterone treatment even in a low-risk group because their tumors would be resistant to ADT but vulnerable to abiraterone. Meanwhile, some men with homozygous wild type in *HSD3B1* gene may be treated with ADT monotherapy even in a high-risk group. However, controversial pharmacological and prognostic significance in therapies using CYP17 inhibitor of genetic polymorphism in *HSD3B1* has recently been reported.^24,25,26^ Almassi et al^24^ have reported improved PFS in variant carrier of *HSD3B1* gene when treated with nonsteroidal CYP17A1 inhibitor ketoconazole. Meanwhile, Hahn et al^25^ failed to show significance in abiraterone treatment, which may be due to statistical underpower. Also, increased serum 3-keto-5α-abiraterone levels were recently observed in variant carriers of *HSD3B1* gene among 30 patients,^26^ where differential enzymatic activity of 5α-reductase among ethnic groups might influence. Then, the value of *HSD3B1* genotype as a predictive marker for ADT and abiraterone for HSPC should be investigated by prospective randomized clinical trials in the future.

In the present study including only Japanese men, the frequency of variant allele (8.7% in metastatic hormone-sensitive prostate cancer and 14.1% in CRPC) appeared to be less than that of the studies from the United States (62.7%, 47.9%,^11^ and 52.9%^12^) but comparable with the Chinese study by Wu et al^22^ (17.5%). This finding suggests less variant frequency in Asian individuals compared with white individuals. Previously, several studies reported the survival of Asian individuals to be better than that of white or black individuals.^27,28,29^ This ethnic difference of *HSD3B1* genetic polymorphism frequency may explain better outcome of ADT in Asian individuals. Meanwhile, according to the results in the current study, the differential frequency of genetic variation in *HSD3B1* may result in distinct oncological outcome among Asian men when treated with abiraterone, which should be investigated in the future.

### Limitations

This study had several limitations, including its retrospective design, relatively small sample size, and lack of abiraterone metabolites measurement. In addition, this study was conducted using the data from multiple institutions, which may have resulted in diagnostic and therapeutic variations among the institutions. Included ethnicity was only Japanese, and abiraterone was used for only CRPC in this study.

## Conclusions

This study confirmed the finding that *HSD3B1* genetic variation may be associated with detrimental outcomes of ADT on prognosis in Japanese men with prostate cancer, augmenting the robustness of previous findings and suggesting universal significance among different ethnicities. Furthermore, opposite prognostic significance in abiraterone treatment was indicated, suggesting promise as a predictive biomarker in ADT and abiraterone including upfront abiraterone therapy for HSPC. However, the present study was limited as described above. Thus, prospective validation studies would be warranted.

## Notes

Supplement. **eTable 1.** Clinicopathological characteristics according to genetic polymorphism in patients treated with primary ADT **eTable 2. **Clinicopathological characteristics according to genetic polymorphism in patients treated with abiraterone
