# Supplementary material for: Association of Missense Polymorphism in HSD3B1 With Outcomes Among Men With Prostate Cancer Treated With Androgen-Deprivation Therapy or Abiraterone
Source: JAMA Netw Open. 2019 Feb 22;2(2):e190115. doi: 10.1001/jamanetworkopen.2019.0115 (PMC6484618; doi:10.1001/jamanetworkopen.2019.0115)
Supplement: Supplement. — eTable 1. Clinicopathological characteristics according to genetic polymorphism in patients treated with primary ADT eTable 2. Clinicopathological characteristics according to genetic polymorphism in patients treated with abiraterone [file jamanetwopen-2-e190115-s001.pdf]

Supplementary Online Content

Shiota M, Narita S, Akamatsu S, et al. Association of missense polymorphism in *HSD3B1* with outcomes among men with prostate cancer treated with androgen-deprivation therapy or abiraterone. *JAMA Netw Open*. 2019;2(2):e190115. doi:10.1001/jamanetworkopen.2019.0115

eTable 1. Clinicopathological characteristics according to genetic polymorphism in patients treated with primary ADT
eTable 2. Clinicopathological characteristics according to genetic polymorphism in patients treated with abiraterone

This supplementary material has been provided by the authors to give readers additional information about their work.

© 2019 Shiota M et al. *JAMA Network Open.*

**eTable 1.** Clinicopathological characteristics according to genetic polymorphism in patients treated with primary ADT					
Gene			*HSD3B1* (rs1047303)		
Variables	All (n = 104)		Homozygous wild-type (n = 95)	Heterozygous and homozygous variant types (n = 9)	P-value
Median age, years (IQR)	72 (67–76)		72 (67–76)	73 (68–75)	0.88
Median PSA at diagnosis, ng/ml (IQR)	244.0 (85.5–744.3)		240.0 (85.2–706.0)	274.0 (81.5–1607.5)	0.51
Biopsy Gleason score, n (%)					
<8	31 (32.6%)		28 (32.6%)	3 (33.3%)	
≥8	64 (67.4%)		58 (67.4%)	6 (66.7%)	0.96
NA	9		9	0	
Clinical stage, n (%)					
cT2/3	65 (72.2%)		57 (69.5%)	8 (100%)	
cT4	25 (27.8%)		25 (30.5%)	0 (0.0%)	0.07
NA	14		13	1	
Clinical stage, n (%)					
N0	40 (44.0%)		39 (47.0%)	1 (12.5%)	
N1	51 (56.0%)		44 (53.0%)	7 (87.5%)	0.06
NA	13		12	1	
Clinical stage, n (%)					
M0	10 (9.6%)		8 (8.4%)	2 (22.2%)	
M1	94 (90.4%)		87 (91.6%)	7 (77.8%)	0.18
Hormonal therapy, n (%)					
Combined androgen blockade	92 (88.5%)		83 (87.4%)	9 (100%)	
Castration	12 (11.5%)		12 (12.6%)	0 (0.0%)	0.26
ADT, androgen-deprivation therapy; IQR, interquartile range; NA, not available					

© 2019 Shiota M et al. *JAMA Network Open.*

**eTable 2.** Clinicopathological characteristics according to genetic polymorphism in patients treated with abiraterone					
Gene			*HSD3B1* (rs1047303)		
Variables	All (n = 99)		Homozygous wild-type (n = 85)	Heterozygous variant type (n = 14)	P-value
Median age at pre-treatment, years (IQR)	74 (67–80)		73 (67–79)	74 (65–81)	0.98
Median PSA at diagnosis, ng/ml (IQR)	77.7 (21.1–327.0)		77.7 (20.1–291.0)	84.7 (24.4–436.0)	0.54
Biopsy Gleason score, n (%)					
<8	14 (15.4%)		12 (15.4%)	2 (15.4%)	
≥8	77 (84.6%)		66 (84.6%)	11 (84.6%)	1.00
NA	8		7	1	
Median PSA at pre-treatment, ng/ml (IQR)	14.7 (4.7–87.1)		17.2 (4.9–93.3)	8.2 (3.8–48.2)	0.18
ECOG PS at pre-treatment, n (%)					
0	65 (65.7%)		55 (64.7%)	10 (71.4%)	
1	26 (26.3%)		23 (27.1%)	3 (21.4%)	
≥2	8 (8.1%)		7 (8.2%)	1 (7.1%)	0.88
Clinical M-stage at pre-treatment, n (%)					
M0	8 (8.1%)		7 (8.2%)	1 (7.1%)	
M1a	10 (10.1%)		7 (8.2%)	3 (21.4%)	
M1b	68 (68.7%)		60 (70.6%)	8 (57.1%)	
M1c	13 (13.1%)		11 (12.9%)	2 (14.3%)	0.49
Prior enzalutamide, n (%)					
Absence	53 (53.5%)		42 (49.4%)	11 (78.6%)	
Presence	46 (46.5%)		43 (50.6%)	3 (21.4%)	0.04*
Prior docetaxel, n (%)					
Absence	58 (58.6%)		48 (56.5%)	10 (71.4%)	
Presence	41 (41.4%)		37 (43.5%)	4 (28.6%)	0.28

© 2019 Shiota M et al. *JAMA Network Open.*

*statistically significant; IQR, interquartile range; PSA, prostate-specific antigen; NA, not available; ECOG, Eastern Cooperative Oncology Group; PS, performance status

© 2019 Shiota M et al. *JAMA Network Open.*
